# Stereotactic body radiotherapy (SBRT) in patients with lung metastases - prognostic factors and long-term survival using patient self-reported outcome (PRO)

**DOI:** 10.1186/s12885-020-6635-8

**Published:** 2020-05-19

**Authors:** Kerstin A. Kessel, Rebekka C. E. Grosser, Kim Melanie Kraus, Hans Hoffmann, Markus Oechsner, Stephanie E. Combs

**Affiliations:** 1 Department of Radiation Oncology, Klinikum rechts der Isar, Technical University of Munich (TUM), Ismaninger Straße 22, 81675 Munich, Germany; 2 Institute of Radiation Medicine (IRM), Helmholtz Zentrum München, Neuherberg, Germany; 3 Deutsches Konsortium für Translationale Krebsforschung (DKTK), Partner Site Munich, Munich, Germany; 4 Division of Thoracic Surgery, Technical University of Munich (TUM), Munich, Germany

**Keywords:** Patient-reported outcome, PRO, Outcomes, Survival, Toxicity, Lung metastasis, Stereotactic body radiotherapy, SBRT

## Abstract

**Objectives:**

The present study aims to evaluate long-term side-effects and outcomes and confirm prognostic factors after stereotactic body radiotherapy (SBRT) of pulmonary lesions. This is the first work that combines the investigated data from patient charts and patient-reported outcome (PRO) up to 14 years after therapy.

**Materials and methods:**

We analyzed 219 patients and 316 lung metastases treated between 2004 and 2019. The pulmonary lesions received a median dose and dose per fraction of 35 Gy (range: 14–60.5 Gy) and 8 Gy (range: 3–20 Gy) to the surrounding isodose. During the last 1.5 years of monitoring, we added PRO assessment to our follow-up routine. We sent an invitation to a web-based survey questionnaire to all living patients whose last visit was more than 6 months ago.

**Results:**

Median OS was 27.6 months. Univariate analysis showed a significant influence on OS for KPS ≥90%, small gross tumor volume (GTV) and planning target volume (PTV), the absence of external metastases, ≤3 pulmonary metastases, and controlled primary tumor. The number of pulmonary metastases and age influenced local control (LC) significantly.

During follow-up, physicians reported severe side-effects ≥ grade 3 in only 2.9% within the first 6 months and in 2.5% after 1 year. Acute symptomatic pneumonitis grade 2 was observed in 9.7%, as grade 3 in 0.5%.

During PRO assessment, 39 patients were contacted, 38 patients participated, 14 participated twice during follow-up. Patients reported 15 cases of severe side effects (grade ≥ 3) according to PROCTCAE classification. Severe dyspnea (*n* = 6) was reported mostly.

**Conclusion:**

We could confirm excellent local control and low toxicity rates. PROs improve and complement follow-up care. They are an essential measure in addition to the physician-reported outcomes. Future research must be conducted regarding the correct interpretation of PRO data.

## Introduction

Lung metastases represent one of the largest groups of metastases. They can be found in more than 50% of cancer patients [1]. Whereas for patients with aggressive metastatic spread, systemic therapy remains the treatment of choice for patients with a low metastatic burden, referred to as oligometastatic (OM) disease, local treatment is favored. Initially, resection was the treatment of choice when technically feasible. In cases where surgery cannot be performed due to the irresectability of the tumor, insufficient medical patient conditions, or patient refusal, stereotactic body radiation therapy (SBRT) reveals a non-invasive alternative treatment [2–6]. Generally, based on the emerging data on SBRT, it can even be considered equieffective as a local treatment alternative to surgery [7]. Keeping in mind that SBRT is non-invasive, quick initiation of systemic treatment is possible.

SBRT delivers a hypofractionated dose to the target volume while sparing surrounding tissue. There have already been published various retrospective studies focusing on local control, survival, and severe toxicities [5, 8–11]. However, the optimal prescription dose still needs further investigation. Former studies have proven that biologically effective doses (BED) of over 105 Gy are required for increased local control rates [7, 12]. When delivering an increased fraction dose to the tumor, the risk of normal tissue toxicity can increase. For patients with severe comorbidities and reduced pulmonary function, SBRT with lower fraction doses might be beneficial, as shown for non-small cell lung cancer (NSCLC) [13].

Outcome predictive factors such as the number and pattern of metastases [14, 15], target volume size [15, 16], a BED over 90 Gy [16], absence of previous systemic therapy [5], no occurrence of new metastases during follow up [17], and an excellent overall condition [13, 16] have been identified in former studies.

Health care profits increasingly by technical advances. For oncology patients, modern documentation methods, such as apps and web-based solutions, support treatment, and follow-up care. Recent studies also showed the effect of increasing overall survival and quality of life [18, 19]. Patient-reported outcome (PRO) is of increasing interested in completing continuous health information [20–22].

The present study aims to evaluate long-term side-effects and outcomes and confirm prognostic factors after SBRT of pulmonary lesions. This is the first work that combines the investigated data from patient charts and PRO up to 14 years after therapy.

## Patients and methods

### Patients

Between 2004 and August 2019, 219 patients and 316 lung metastases were treated consecutively at the Department of Radiation Oncology at the Klinikum rechts der Isar, Munich. Exclusion criteria were radiotherapy (RT) with a simultaneously integrated boost, radiosurgery, or early termination of treatment. Data was collected retrospectively and documented in the institutional database. The local ethics committee of the Medical Faculty of the Technical University München (TUM) approved the study (vote numbers: 257/16, 438/16).

The median age at SBRT was 68 years (range 6–91). Gender distribution was about 3:2 (male:female), see patient characteristics (Table 1). Of all, 62 (19.6%) patients had multiple SBRTs of pulmonary metastases.

**Table 1:** Patient characteristics of 219 patients and 316 lesions

	n	%	Median (range)
Gender			
Female	90	41.1	
Male	129	58.9	
Age at RT [years]			68 (6–91)
Primary tumor type			
NSLC	56	17.7	
CRC	93	29.4	
Melanoma	11	3.5	
Breast Cancer	20	6.3	
Others	136	43.0	
Number of pulmonary METs			
≤ 3	203	64.2	
> 3	113	35.8	
MET location			
Central	34	10.8	
Upper lobe	130	41.1	
Middle lobe	23	7.3	
Lower lobe	129	40.8	
Laterality			
Right	169	53.5	
Left	145	45.9	
Bilateral	2	0.6	
Extra thoracic MET			
Yes	131	41.5	
No	185	58.5	
KPS [%] at RT			
100	8	2.5	
90	141	44.6	
80	137	43.4	
70	22	7.0	
60	7	2.2	
50	1	0.3	
Prior thorax RT			
Yes	87	27.5	
No	229	72.5	
CHT between MET diagnosis and SBRT			
Yes	58	18.4	
No	258	81.6	
Time primary diagnosis until MET diagnosis [months]			30.9 (0–265.9)
Time MET diagnosis until RT [months]			1.7 (0–127.3)
PTV [ml]			28.8 (2.0–517.0)
GTV [ml]			5.4 (0.1–217.6)

*NSCLC* Non-small cell lung cancer, *CRC* Colorectal carcinoma, *KPS* Karnofsky performance score, *RT* Radiotherapy, *CHT* Chemotherapy, *MET* Metastasis, *PTV* Planning target volume, *GTV* Gross tumor volume

### Treatment planning and radiotherapy

All patients received SBRT. Until 2015, vacuum couch and low-pressure foil (Medical Intelligence GmbH, Schwabmünchen, Germany) ensured immobilization. Since then, abdominal compression was used. When needed, further movement reduction was achieved by administering oxygen to the patient or using abdominal pressure. Before 2010, patients received a conventional computed tomography (CT) for treatment planning, since then 4D-CT were acquired additionally to account for tumor motion adequately. Setup verification was accomplished by onboard cone-beam CT (CBCT) before irradiation.

For all treatment concepts, both organs at risk (OAR) and gross tumor volume (GTV) were defined in the contrast-enhanced treatment planning CT. In 30 (9.5%) cases, positron emission tomography (PET) imaging was performed additionally. The planning target volume (PTV) resulted from the GTV with an additional margin of 5–10 mm. In cases where a 4D-CT was acquired the tumor (GTV) was delineated in all breathing phases resulting in an internal target volume (ITV); the PTV resulted then from the ITV + 5 mm. Additionally, a margin up to 5 mm was added, resulting in the PTV. SBRT was delivered with a Clinac Trilogy linear accelerator (Varian Medical Systems, Palo Alto, CA, USA). The pulmonary lesions were treated with a median dose and dose per fraction of 35 Gy (range: 14–60.5 Gy) and 8 Gy (range: 3–20 Gy) to the surrounding isodose.

For better comparison of the transmitted doses, we calculated the BED_10iso_ and BED_10PTVmean_ doses, assuming an a/ß of 10. BED_10iso_ is calculated from the 60% isodose level, BED_10PTVmean_ from the mean PTV dose. The median BED_10iso_ was 33.0 Gy (range: 11.2–126.0); the median BED_10PTVmean_ dose was 106.7 Gy (range: 29.1–258.9).

### Follow-up and patient-reported outcome (PRO)

Patients were enrolled in a tight follow-up regimen with the first follow-up after 6–8 weeks, including contrast-enhanced CT as well as clinical assessment. Further examinations were scheduled after 3 months during the first year, and in 6 to 12-months intervals thereafter depending on the prognosis or as clinically needed. All decisions about further therapies were made on an interdisciplinary basis. Symptoms and toxicity before SBRT and during follow-up were graded according to CTCAE scores (Common Terminology Criteria for Adverse Events, Version 4.03). Depending on their time of occurrence, symptoms were assigned to one of the following observation intervals: acute symptoms (up to 6 months after SBRT), late symptoms (6–24 months after SBRT), and long-term symptoms (> 2 years after SBRT). To complete long-term follow-up, we acquired PRO via web, mail, or telephone. The questions focused on current side effects after SBRT and the actual medical condition, including worsening or improvement of symptoms. We used the German items of PROCTCAE™ (Patient-reported Outcome for Common Terminology Criteria for Adverse Events), developed by the NCI (National Cancer Institute) [17]. Also, information about recent imaging results and further treatment procedures after SBRT were reported. To avoid bias and remembering problems, we explicitly asked for incidences during the last week, as Mendoza et al. suggested [23].

During the last 1.5 years of monitoring, we added PRO assessment to our follow-up routine. We sent an invitation to a web-based survey questionnaire to all living patients whose last visit was more than 6 months ago (*n* = 39). Patients living abroad were not contacted. If a patient did not submit the online survey, a paper-based version of the questionnaire was sent via mail. If patients also did not respond to the letter, we phoned them and inquired them personally based on the questionnaire.

### Statistics

Statistical calculations were performed using SPSS Statistics v25 (IBM, USA). If patients received multiple treatments, only the first treatment (*n* = 219) was used for the calculation of overall survival (OS) and distant progression (DP). DP was defined as progression outside the lung. Local control (LC) was calculated for all lesions (*n* = 316). OS was calculated from the last treatment day until death or last follow-up; DP/LC from the last treatment day until the date of distant/local progression or until death or last follow-up.

OS, DP, and LC analyses were based on the Kaplan–Meier method using the log-rank test; Cox regression was used for univariate and multivariate analyses. The following prognostic factors were analyzed: age, gender, Karnofsky Performance Score (KPS), GTV, PTV, PET imaging before treatment, previous chemo, previous external irradiation, number of pulmonary metastases, absence of extrathoracic metastases, controlled primary tumor, primary tumor type, as well as chemotherapy (CHT) between diagnosis of lung metastases and SBRT. We used receiver operating characteristic (ROC) statistics to determine thresholds for grouped variables. A *p*-value < 0.05 was considered as statistically significant. For patients with multiple follow-ups during one observation interval, we counted the highest CTCAE grade of symptoms/side effects and the lowest KPS.

## Results

### Outcomes

Table 2 provides data on OS, DP, and LC. Median follow-up was 16.5 months (range: 0–14.5 years). Median OS was 27.6 months (95% Confidence Interval (CI): 22.8–32.4), median DP was 17.4 months (95% CI: 9.2–25.6), mean LC was 135.1 months (95% CI: 123.5–146.7) as median was not reached.

**Table 2:** Life table for OS, DP, LC

	Proportion surviving after					
	1-year	2-year	3-year	4-year	5-year	10-year
OS	74%	54%	39%	29%	26%	17%
DP	58%	46%	40%	37%	34%	32%
LC	92%	84%	78%	77%	75%	75%

*OS* Overall survival, *DP* Distant progression, *LC* Local control

### Prognostic factors

Univariate analysis (Table 3) showed a significant influence on OS for several prognostic factors. KPS < 90% was associated with a lower OS (*p* < 0.001), whereas a small GTV (*p* = 0.002) and PTV (*p* = 0.003) showed a significant impact for better OS. The absence of external metastases (*p* = 0.020), ≤3 pulmonary metastases (*p* = 0.014), and controlled primary tumor (*p* < 0.001) also correlate with longer survival. Patients that received higher doses (BED_10iso_: *p* = 0.001, and BED_10PTVmean_: *p* = 0.007) and no chemotherapy between diagnosis and SBRT (*p* = 0.047) lived longer.

**Table 3:** *P*-values of univariate and multivariate analyses for OS, DP, and LC. Multivariate analysis was performed with the significant items of univariate analysis

	OS (*n* = 219)		DP (*n* = 219)		LC (*n* = 316)	
	UVA	MVA	UVA	MVA	UVA	MVA
Age at RT°	0.667	–	0.630	–	0.003*	0.320
Gender	0.401	–	0.092	–	0.220	–
KPS (< 90 vs. ≥90%)	< 0.001*	< 0.001*	< 0.001*	< 0.001*	0.844	–
Primary tumor type (NSCLC vs CRC vs. Melanoma vs. Breast)	0.985	–	0.954	–	0.437	–
GTV°	< 0.002*	0.439	0.035*	0.919	0.762	–
GTV (< 8 vs ≥8 ml)	< 0.001*	–	0.005*	–	0.844	–
PTV°	0.003*	0.904	0.046*	0.599	0.211	–
PTV (< 35 vs ≥35 ml)	< 0.001*	–	0.017*	–	0.842	–
Planning PET-CT	0.264	–	0.765	–	0.133	–
Number of pulmonary METs (≤3 vs. > 3)	< 0.001*	0.014*	0.078	–	< 0.001*	< 0.001*
Absence extra thoracic MET	< 0.001*	0.020*	< 0.001*	< 0.001*	0.859	–
Controlled primary tumor	< 0.001*	< 0.001*	0.001*	0.003*	0.632	–
CHT between MET diagnosis and SBRT	0.047*	0.176	0.468	–	0.097	–
BED_10iso_°	0.001*	0.289	0.413	–	0.429	–
BED_10PTVmean_°	0.007*	0.468	0.439	–	0.955	–
Time from primary diagnosis to MET diagnosis°	0.090	–	0.985	–	0.882	–
Time from primary diagnosis to MET diagnosis (< 12 vs. ≥12 months)	0.884	–	0.291	–	0.336	–

*UVA* Univariate analysis, *MVA* Multivariate analysis, *OS* Overall survival, *DP* Distant progression, *LC* Local control, *KPS* Karnofsky Performance Score, *RT* Radiotherapy, *CHT* Chemotherapy, *MET* Metastasis, *PTV* Planning target volume, *GTV* Gross tumor volume, *NSCLC* non-small cell lung cancer, *CRC* colorectal carcinoma, * significant *p*-value, *°* continuous variable

In the multivariate analysis, KPS, ≤3 pulmonary metastases, absence of external metastases, and controlled primary remained significant for OS.

For DP, KPS ≥90% (*p* < 0.001), a small GTV (*p* = 0.035) and PTV (*p* = 0.046), the absence of external metastases (*p* < 0.001), and a controlled primary tumor (*p* = 0.003) were associated with a longer distant progression-free survival.

The multivariate analysis proved KPS, a controlled primary tumor, and the absence of external metastases as significant factors.

Patients with ≤3 pulmonary metastases (*p* < 0.001) had a higher LC rate. Also, the age (*p* = 0.003) influenced LC significantly.

In multivariate analysis, only ≤3 pulmonary metastases remained significant.

### Toxicity and PRO

Documented toxicity was mainly graded 1 or 2 (Tables 4 and 5). During follow-up, physicians reported severe side-effects ≥ grade 3 in only 2.9% (6/207) within the first 6 months and in 2.5% (3/119) after 1 year. Acute symptomatic pneumonitis grade 2 was observed in 9.7% (20/207), as grade 3 in 0.5% (1/207). Only new and worsened symptoms after RT are listed in Table 5.

**Table 4:** Physician-reported new and worsened side effects during follow-up according to CTCAE classification. PRO data is reported as PROCTCAE and listed separately, hence, not included in the physician reported results

CTCAE grade	Pre RT (*n* = 219)				< 6 months (*n* = 208)				6–12 months (*n* = 130)				> 12 months (*n* = 120)				PRO (*n* = 38)			
	1	2	3	4	1	2	3	4	1	2	3	4	1	2	3	4	1	2	3	4
Pain	6 (3%)	4 (2%)	–	–	14 (7%)	4 (2%)	1 (< 1%)	–	6 (5%)	2 (2%)	–	–	3 (3%)	6 (5%)	1 (1%)	–	9 (24%)	7 (18%)	–	–
Fatigue	2 (1%)	–	–	–	21 (10%)	5 (2%)	–	–	4 (3%)	2 (2%)	–	–	5 (4%)	3 (3%)	–	–	9 (24%)	16 (42%)	–	–
Nausea/vomiting	–	–	–	–	1 (< 1%)	–	–	–	–	–	–	–	1 (1%)	–	–	–	1 (3%)	3 (8%)	–	–
Dermatitis	–	–	–	–	3 (1%)	–	–	–	1 (1%)	–	–	–	–	1 (1%)	–	–	3 (8%)	1 (3%)	1 (3%)	–
Hyperpigmentation	–	–	–	–	3 (1%)	–	–	–	2 (2%)	–	–	–	–	–	–	–	2 (5%)	–	–	–
Edema	1 (< 1%)	–	–	–	6 (3%)	–	–	–	5 (4%)	2 (2%)	–	–	3 (3%)	1 (1%)	–	–	7 (18%)	7 (18%)	–	–
Osteonecrosis, rib fracture	–	–	–	–	–	–	–	–	–	–	–	–	–	–	1 (1%)	–	–	–	–	–
Sensory deficits	–	1 (< 1%)	–	–	–	–	–	–	–	–	–	–	2 (2%)	–	–	–	10 (26%)	5 (13%)	1 (3%)	1 (3%)
Motor deficits	–	–	–	–	2 (1%)	–	–	–	–	–	–	–	2 (2%)	–	–	–	7 (18%)	6 (16%)	2 (5%)	–
Weight loss	–	–	1 (< 1%)	–	3 (1%)	–	–	–	3 (2%)	–	–	–	5 (4%)	–	–	–	4 (11%)	5 (13%)	1 (3%)	–
Fibrosis	1 (< 1%)	–	–	–	25 (12%)	3 (1%)	–	–	22 (17%)	6 (5%)	–	–	13 (11%)	2 (2%)	1 (1%)	–	–	–	–	–
Pneumonitis	–	–	–	–	24 (12%)	20 (10%)	1 (< 1%)	–	5 (4%)	3 (1%)	–	–	1 (1%)	1 (1%)	–	–	–	–	–	–
Dysphagia	2 (1%)	2 (1%)	1 (< 1%)	–	3 (1%)	–	1 (< 1%)	–	1 (1%)	2 (2%)	–	–	4 (3%)	–	–	–	10 (26%)	4 (11%)	1 (3%)	–
Dyspnea	17 (8%)	9 (4%)	1 (< 1%)	–	27 (13%)	23 (11%)	3 (1%)	–	5 (4%)	3 (2%)	–	–	7 (6%)	6 (5%)	–	–	10 (26%)	8 (21%)	4 (11%)	2 (5%)
Cough	15 (7%)	1 (< 1%)	–	–	35 (17%)	5 (2%)	–	–	10 (8%)	2 (2%)	–	–	15 (13%)	2 (2%)	–	–	10 (26%)	14 (37%)	1 (3%)	–
Xerostomia	2 (1%)	–	–	–	3 (1%)	1 (< 1%)	–	–	–	–	–	–	1 (1%)	1 (1%)	–	–	11 (29%)	11 (29%)	–	–
Dysphonia	1 (< 1%)	–	–	–	1 (< 1%)	–	1 (< 1%)	–	2 (2%)	–	–	–	1 (1%)	–	–	–	8 (21%)	4 (11%)	1 (3%)	–

*RT* Radiotherapy, *PRO* Patient-reported outcome, *CTCAE* Common Terminology Criteria for Adverse Events

**Table 5:** Comparison of physician-reported outcome after RT and PRO (*n* = 38). In both groups, only new and worsened symptoms after RT are listed

CTCAE grade	Physician-reported after RT			PRO		
	1/2	3	4	1/2	3	4
Pain	9 (24%)	–	–	16 (42%)	–	–
Fatigue	10 (26%)	–	–	25 (66%)	–	–
Nausea/vomiting	1	–	–	4 (11%)	–	–
Dermatitis	1 (3%)	–	–	4 (11%)	1 (3%)	–
Hyperpigmentation	2 (5%)	–	–	2 (5%)	–	–
Edema	4 (11%)	–	–	14 (37%)	–	–
Osteonecrosis, rib fracture	–	–	–	–	–	–
Sensory deficits	–	–	–	15 (39%)	1 (3%)	1 (3%)
Motor deficits	–	–	–	13 (34%)	2 (5%)	
Weight loss	4 (11%)	–	–	9 (24%)	1 (3%)	–
Fibrosis	17 (45%)	–	–	–	–	–
Pneumonitis	10 (26%)	–	–	–	–	–
Dysphagia	2 (5%)	–	–	14 (37%)	1 (3%)	–
Dyspnea	16 (42%)	–	–	18 (47%)	4 (11%)	2 (5%)
Cough	14 (37%)	–	–	24 (63%)	1 (3%)	–
Xerostomia	1 (3%)	–	–	22 (58%)	–	–
Dysphonia	–	–	–	12 (32%)	1 (3%)	–

*RT* Radiotherapy, *PRO* Patient-reported outcome, *CTCAE* Common Terminology Criteria for Adverse Events

At the time of analysis, 153 (153/219, 69.9%) patients treated with SBRT were deceased, 15 (15/219, 6.8%) were seen during regular follow-up visits, and 12 (12/219, 5.5%) were living abroad and lost-to-follow-up. The remaining 39 (39/219, 17.8%) were contacted for PRO assessment. Of the 39 patients, 38 (38/39, 97.4%) participated in the PRO assessment; 14 participated twice during follow-up. The median time between SBRT and PRO was 53.2 months (range: 2.1 months - 14.5 years).

**Fig. 1:**
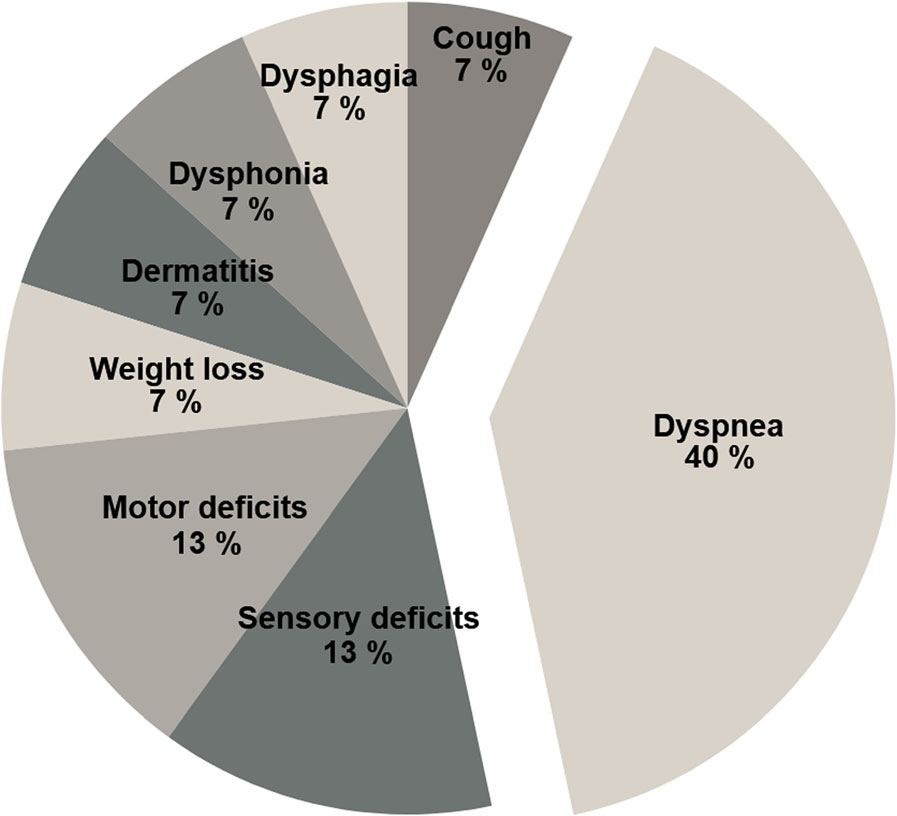
Chart of all severe side effects reported by PRO (*n* = 15)

Patients reported 15 cases of severe side effects grade ≥ 3 during PRO assessment (Fig. 1, Table 4). All patients with severe dyspnea (*n* = 6) were diagnosed with chronic obstructive pulmonary disease before SBRT. Four of these were also frequent smoker in the past, one was non-smoker, one had an unknown smoking history. The patient with dysphonia grade 3 had a squamoid carcinoma of the larynx. Out of all, four and three severe side effects were reported each by one single patient.

## Discussion

In our analysis, we evaluated outcomes after high-precision SBRT of 316 lung metastases and determined long-term results, including PRO. We could demonstrate that our rates of LC after SBRT of pulmonary lesions and the low rate of severe toxicity are comparable to those found in the literature [5, 9, 16, 24–26].

Previously, our group demonstrated prognostic factors in 129 lung cases [27]. The present work comprises a larger group of patients with SBRT treatment and, in particular, the inclusion of PRO assessment. With an LC rate of 78% after 3 years, we present excellent treatment results known for high-precision SBRT. Also, we could show a very beneficial risk-benefit profile of SBRT among our patient cohort. The evaluation of PRO enabled us to collect comprehensive information about symptoms of patients up to 14 years after SBRT. This distinguishes our work from other studies investigating comparable patient groups with pulmonary metastases.

The OS of the present study is lower compared to other studies and may be explained by applied exclusion criteria. De Rose, for example, only analyzed oligometastatic patients with controlled primary tumor [9] (3-year survival rate: 64% vs. this study: 39%). Navarria et al. excluded patients whose number of metastatic sites was > 5 and who had a short progression-free survival (3-year survival rate 73% vs. this study: 39%) [8].

Inoue et al. also achieved comparably low 2- and 3-year OS rates of 47 and 32%, respectively. They also evaluated a quite unselected group of patients [28]. Kang et al. showed a five-year OS rate of 29% [29], which is higher compared to 26% reported in the present study. However, they excluded patients with metastases in more than one organ.

We showed a benefit in OS for patients with a KPS ≥90%, small GTV and PTV, higher BED doses, absence of external metastases, ≤3 pulmonary metastases, and a controlled primary tumor. This prognostic impact could also be shown by other studies [6, 7, 25, 27, 30, 31].

Of all patients, 9.6% (20/208) developed acute pneumonitis grade 2, one (0.5%) had to be hospitalized (grade 3). Late pneumonitis occurred in 6.2% (8/130) and in 1.6% (2/120) > 12 months after RT. One patient that was treated with RT of the esophagus and regional lymphatic pathways suffered from myocardial infarction, which was treated with PCI (percutaneous coronary intervention). Another patient that received a total dose of 35 Gy in 5 fractions had grade 3 osteoradionecrosis 2.5 years after SBRT. Those toxicity rates are comparable to others [28, 32–34].

Dyspnea after SBRT was quite a common complaint (25.6%, 53/208). However, this is most likely not associated with RT, since many of these patients did not undergo surgery due to chronic obstructive pulmonary disease (COPD), respiratory insufficiency, or an insufficient heart because of other reasons. Smoking was not regularly documented.

PRO is a measure that becomes more important in oncological treatment. In a study with primary lung cancer patients, Denis et al. [18] showed that regularly reported symptoms by the patient leads to a 6-month prolonged OS. Similar results were reported by Basch et al. [19]. In a randomized study with 766 patients, the experimental arm reported PRO data regularly, which also resulted in an improved OS.

As in all retrospective analyses, a limitation is the heterogeneous and poor documentation of clinical data during follow-up. Precisely for this reason, conducting PRO is a relevant addition to regular clinical assessment and leads to the higher completeness of data [35–37]. Especially in cases where long-term side effects of treatment are interesting, and patients are easily lost to follow-up, PRO is an efficient method to quantify symptomatic improvement or worsening [20, 21].

When interpreting PRO data, it needs to be considered that the underlying causes of occurring symptoms could not be investigated explicitly. Many cancer patients had lots of different treatments, and side effects cannot be attributed to one therapy. Particularly in cohorts of older patients, this might lead to an increase in reported symptoms in the long-term follow-up due to rising numbers of comorbidities [20]. Another challenge is that symptoms that occurred before treatment but are remembered later when the patients fill out the questionnaire can also lead to misinterpretation. During the PRO assessment, patients often reported higher rates of grade 3 and 4 toxicities. As mentioned earlier, half of these complaints were declared by only two patients indicating the reasonable suspicion of personal sensitivities.

Furthermore, in a regular follow-up visit, physicians usually do not ask patients for all possible side effects; they usually concentrate on specific relevant side effects due to limited time. Besides, the PRO patients had a web survey or paper questionnaire at home where they did not have any time pressure to remember but could rethink about their symptoms several times. Questionnaires and web surveys suggest various complaints, so that patients may be more likely to cross more than they would think about in a personal anamnesis. Physicians also might tend to filter the patient information and only document treatment toxicities and not everything the patient reported.

The PRO-CTCAE is a standardized classification of symptom measurement, however, the limitation of a direct translation of the patient-reported to a physician-reported symptom remains [38]. It appears that subjective personal sensitivity has a relevant influence on how patients fill out questionnaires. Researchers of the University of Manchester found that toxicity grades of patients with lung RT differ compared to physician-reported results in up to one grade, mostly because patients have a more pessimistic view on their symptoms [39]. The patient-reported view has a higher correlation with the quality of life than physician-documented toxicity [39].

The other aspect one needs to bear in mind when interpreting or comparing the data is that the PRO measures were not taken parallel to physician measures. PRO assessment was additionally conducted when patients were no longer participating in the clinical follow-up workflow to gather information on a missing health status.

A further limitation in our study is that the PRO assessment was only conducted in 17.8% (39/219); most patients treated with SBRT (69.9%) were already diseased. However, the response rate of our contacted patients was very high, with 97.4% (38/39). If implemented earlier or even before therapy, the results would be more expressive. Conduction of PRO and clinical assessment at the same time would have increased the comparability of patient-reported and physicians-reported data. Other studies with a prospective approach detected promising benefits of PRO assessment [18, 19].

For implementing PRO in clinical routine, it is necessary to develop clear standards for analysis and interpretation. Eventually, parameters, such as necrosis, fibrosis, and pneumonitis need medical imaging or pathological investigation for diagnosis, especially when in a low stage. A questionnaire or interview will not replace imaging; however, PRO data might indicate whether earlier imaging is necessary.

## Conclusion

SBRT is an effective treatment of pulmonary metastases. We could confirm excellent local control and low toxicity rates. PROs improve and complement follow-up care. They are an essential measure in addition to the physician-reported outcomes and should be permanently included in the follow-up workflow. Future research must be conducted regarding the correct interpretation of PRO data. Eventually, the integration of regular PRO into clinical routine combined with high-end RT treatment will further improve patient outcomes.

## Data Availability

The datasets used and/or analyzed during the current study are available from the corresponding author on reasonable request.
